# Cost effectiveness of outpatient treatment for febrile neutropaenia in adult cancer patients

**DOI:** 10.1038/bjc.2011.101

**Published:** 2011-04-05

**Authors:** O Teuffel, E Amir, S Alibhai, J Beyene, L Sung

**Affiliations:** 1 Division of Haematology/Oncology, Child Health Evaluative Sciences, The Hospital for Sick Children, 555 University Avenue, Toronto, Ontario, Canada M6G 1X8; 2 Division of Medical Oncology, Princess Margaret Hospital, 610 University Avenue, Toronto, Ontario, Canada M5G 2M9; 3 Department of Medicine, University Health Network, 200 Elizabeth Street, Toronto, Ontario, Canada M5G 2C4; 4 Dalla Lana School of Public Health, University of Toronto, 27 King's College Circle, Toronto, Ontario, Canada M5S 1A1

**Keywords:** cost effectiveness, fever, neutropaenia, ambulatory care, in-patients

## Abstract

**Background:**

There is uncertainty whether low-risk episodes of febrile neutropaenia (FN) in adult cancer patients are best managed in the in- or outpatient setting.

**Methods:**

A Monte Carlo cost–utility model was created to compare four treatment strategies for low-risk FN: (1) treatment in hospital with intravenous antibiotics (HospIV); (2) early discharge after 48 h in-patient observation, followed by oral outpatient treatment (EarlyDC); (3) outpatient management with IV antibiotics (HomeIV); and (4) outpatient management with oral antibiotics (HomePO). The model used a health-care payer perspective and a time horizon of one FN episode. Outcome measures were quality-adjusted FN episodes (QAFNE), costs (Canadian dollars) and incremental cost-effectiveness ratios (ICER). Parameter uncertainty was assessed with probabilistic sensitivity analyses.

**Results:**

HomePO was cost saving ($3470 *vs* $4183), but less effective (0.65 QAFNE *vs* 0.72 QAFNE) than HomeIV. The corresponding ICER was $10 186 per QAFNE. Both EarlyDC ($6115; 0.66 QAFNE) and HospIV ($13 557; 0.62 QAFNE) were dominated strategies. At a willingness-to-pay (WTP) threshold of $4 000 per QAFNE, HomePO and HomeIV were cost effective in 54 and 38% of simulations, respectively.

**Interpretation:**

For adult cancer patients with an episode of low-risk FN, treatment in hospital is more expensive and less effective than outpatient strategies.

Despite recent advances in infection prevention, febrile neutropaenia (FN) remains a frequent complication of chemotherapy in adult cancer patients (Renwick *et al*, 2009). For many decades, owing to the potential for life-threatening sepsis, the standard treatment of FN had been in-patient management with broad-spectrum intravenous (IV) antibiotics for all patients (Hughes *et al*, 2002). It is now well recognised, however, that adult patients with FN are a heterogeneous population, with only a small proportion developing serious medical complications (Klastersky *et al*, 2000). Consequently, current national and international guidelines have endorsed less aggressive empiric antibiotic strategies, including outpatient and/or oral antibiotic regimens, for adult cancer patients with low-risk FN (Tamura, 2005; Segal *et al*, 2008). However, there remains uncertainty with regard to whether low-risk FN in adult cancer patients is best managed in-hospital or in an outpatient setting. Some of this uncertainty is related to questions as to whether there are differences in the safety or efficacy of the different management strategies. Thus far, no study has quantitatively synthesised the evidence comparing outpatient *vs* in-patient management. Additional uncertainty is related to health-related quality of life and preferences of patients in the context of FN (Brown *et al*, 2001; Havrilesky *et al*, 2009). In contrast, there is a strong body of evidence published in the literature suggesting that costs of in-hospital treatment are greater than the costs of ambulatory care for FN (Bennett and Calhoun, 2007; Liou *et al*, 2007). A major limitation of current data is whether higher costs of in-patient care can be justified on the basis of safety and efficacy issues, or patients' preferences. Clarifying this issue may have major resource implications given the relative frequency and costs associated with admission to hospital with FN.

Therefore, the objective of this study was to construct a cost–utility model to determine the optimal treatment strategy for low-risk FN in adults with cancer. In this study, costs and effectiveness (measured as quality-adjusted FN episodes (QAFNE)) of four different treatment strategies for low-risk FN were examined. These included entire in-patient management, treatment at home after an initial observation in hospital, entire outpatient management with IV antibiotics and entire outpatient management with oral antibiotics.

## Materials and methods

### Overview

Adult cancer patients with low-risk FN were entered into a decision-analytic model as a hypothetical cohort. The decision tree model encompassed one episode of FN. A simple decision tree model was chosen rather than a state-transition model (Markov model) for two reasons: (1) the concept of changing transition probabilities between different health states did not apply to our clinical scenario (acute health state); (2) there are no data in the literature indicating that long-term implications of a single low-risk episode differ between strategies (Rubenstein *et al*, 1993; Malik *et al*, 1995; Hidalgo *et al*, 1999; Minotti *et al*, 1999; Rapoport *et al*, 1999; Innes *et al*, 2003; Sebban *et al*, 2008). Death was not included in the model, because this is a very unusual event in low-risk FN as described in the literature. The rare cases of death reported from randomised controlled trials (RCTs) appear to be randomly distributed between in- and outpatient settings (Malik *et al*, 1995; Hidalgo *et al*, 1999; Rapoport *et al*, 1999; Innes *et al*, 2003). Furthermore, there has not been a single case of death related to infection in any observational study of outpatient management reported within the last 20 years (Talcott *et al*, 1994; Escalante *et al*, 2004; Chamilos *et al*, 2005; Klastersky *et al*, 2006; Rolston *et al*, 2006). Thus, inclusion of death would not substantially contribute to preferences for any one strategy. Consequently, effectiveness was measured as QAFNE rather than quality-adjusted life years (QALYs), which is the preferred outcome measure in state-transition models (Siegel *et al*, 1996).

### Decision-analytical model

**Figure 1:**
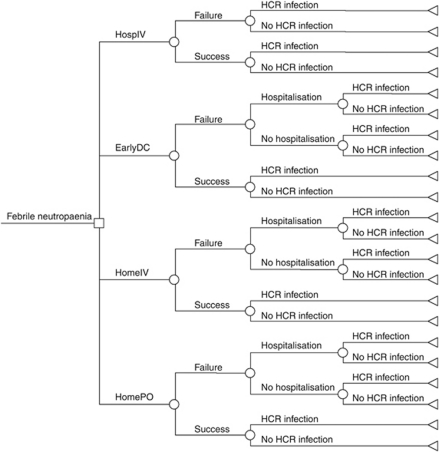
Clinical decision model to compare different treatment strategies for low-risk febrile neutropaenia in adult cancer patients; four treatment strategies are evaluated: (1) entire HospIV; (2) EarlyDC; (3) HomeIV; and (4) HomePO. HCR indicates health-care-related infection.

A decision-analytic model was constructed using TreeAge Pro 2009 and examined four strategies: (1) entire treatment in hospital with IV antibiotics (HospIV); (2) early discharge consisting of 48 h in-patient observation with IV antibiotics, followed by oral outpatient treatment (EarlyDC); (3) entire outpatient management with IV antibiotics (HomeIV); and (4) entire outpatient management with oral antibiotics (HomePO). Piperacillin/tazobactam plus tobramycin was chosen as first-line IV antibiotics, whereas oral treatment consisted of ciprofloxacin plus amoxicillin/clavulanate (Hughes *et al*, 2002). Consistent with the published literature, average treatment duration was defined as 6 days for all four strategies (Rubenstein *et al*, 1993; Rapoport *et al*, 1999; Innes *et al*, 2003; Sebban *et al*, 2008). Outcome measures were QAFNE, costs (in Canadian dollars) and incremental cost-effectiveness ratios (ICER). The structure of the decision tree and its pathways are illustrated in Figure 1. Further details of the decision-analytic model are outlined in Supplementary data A.

### Probabilities

A systematic review was conducted to obtain the best available evidence with regard to outpatient management of FN (only RCTs were considered) (Teuffel *et al*, 2011). An electronic search of OVID MEDLINE from 1950 to February 2010, EMBASE from 1980 to February 2010 and The Cochrane Central Register of Controlled Trials (first quarter of 2010) was performed. Conference Proceedings from 2007 to 2010 were retrieved from ISI Web of Science and SCOPUS. From 1448 reviewed articles, seven studies met pre-defined inclusion criteria (Rubenstein *et al*, 1993; Malik *et al*, 1995; Hidalgo *et al*, 1999; Minotti *et al*, 1999; Rapoport *et al*, 1999; Innes *et al*, 2003; Sebban *et al*, 2008). ‘Low risk’ was defined as per study protocol.(Teuffel *et al*, 2011) The mean rates of treatment failure and hospital readmission were extracted from the seven studies stated above (Table 1). The time frame for events as reported by included studies encompassed 30 days. The range of possible outcomes was estimated from studies describing the most extreme results (Table 1). The probability of health-care-related infections was obtained from several observational studies (Simon *et al*, 2000, 2008; Kamboj and Sepkowitz, 2009).

### Utilities

Health utilities reflect quantitative assessments of the strength of individual preferences for health states when measured under conditions of uncertainty (VonNeumann and Morgenstern, 1953; Torrance, 1987). We elicited preferences from 77 patients receiving active treatment for cancer at Princess Margaret Hospital (PMH, Toronto, Canada), with regard to the four different treatment strategies. A current or previous episode of FN was not mandatory for inclusion. Hypothetical scenarios were presented (Supplementary data B) and a visual analogue scale (VAS) was used to measure patients' preferences for the four different health states (strategies) (Patrick *et al*, 1973; Drummond *et al*, 2005). Respondents were asked to mark their preferences on a horizontal 10-cm line anchored at the left end by the worst possible health or death (score of 0) and at the right end by perfect health (score of 1). Visual analogue scale scores are not usually considered utilities (because they are measured under conditions of certainty), but they are related to standard gamble utilities in a nonlinear manner and can be transformed to the latter using a power function (Torrance *et al*, 2001). The following conversion algorithm was used in this study for deriving a standard gamble score (Table 1): 1−(1−VAS)^1.61^ (Torrance, 1976; Torrance *et al*, 2001). Utility deductions for treatment failure, readmission and health-care-related infection were applied (Table 1) to each of the four treatment options to arrive at final utilities for all 22 possible health states (Figure 1). Effectiveness (QAFNE) was derived from the weighted average products of health state probabilities and health state utilities for each pathway.

### Costs

The analysis adopted the health-care payer's perspective in Canada and included all relevant direct health costs. Cost factors associated with hospitalisation, initial consultation, outpatient visits, home care nursing and medications were obtained from the following sources: (1) Ontario Health Insurance Schedule of Benefits and Fees; (2) local finance offices at PMH (hospital fees/charges and home care nurse visits); and (3) the Department of Pharmacy at PMH (drug costs). The following range was applied to the probabilistic analyses: lower limit=baseline estimate × 0.5 and upper limit=baseline estimate × 2 (Table 1). Detailed information about cost factors are given in Supplementary data C. All costs are quoted in 2009 Canadian dollars. As all outcomes occurred within one episode of FN, no discounting was applied.

### Probabilistic sensitivity analysis

A probabilistic sensitivity analysis was performed to address the joint uncertainty of all model parameters simultaneously and to provide a more accurate estimate of the average ICER. We performed 10 000 second-order Monte Carlo simulations and reported results in two ways. First, we reported mean health outcomes and costs with 95% confidence intervals, that is, the range covered by 95% of our simulations. Second, we graphed results on a cost-effectiveness acceptability curve, which starts from the assumption that cost-effectiveness ratios are considered attractive if the estimates are less than the amount society would be willing to pay for an additional unit gain in health. The curve was generated that used net benefits to graph the changing percentage of simulations for which each comparator is cost effective relative to all other strategies.

As a secondary analysis, all variables were tested in a one-way (plausible range) deterministic sensitivity analysis.

For all analyses, the willingness-to-pay (WTP) threshold was set at $4000 per QAFNE, with a range testing from $0 to $20 000 for the acceptability curve. We assumed that one episode of FN did not exceed 1 month (time frame of event probabilities=30 days). Hence, $4000 per QAFNE was used as baseline value as this approximates a WTP threshold of $50 000 per QALY, a threshold commonly used in health economic evaluations (Shiroiwa *et al*, 2010).

## Results

### Base–case analysis

**Figure 2:**
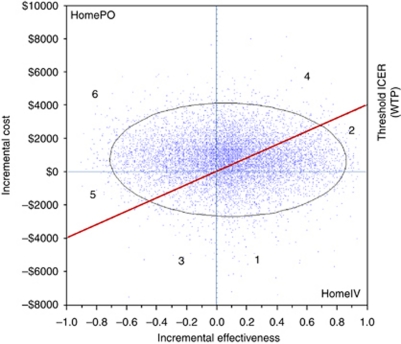
The ICE scatter plot includes a single set of points representing pairs of incremental cost and effectiveness values from the simulation results (*n*=10 000) relative to a baseline (oral treatment at home; HomePO). The comparator in this scatter plot is IV treatment at home (HomeIV). The slope intersecting the *y* axis at $4000 displays the WTP threshold. In addition to the WTP line, a 95% confidence ellipse is drawn in the ICE scatter plot. The graph can be divided into several distinct regions: (1) HomeIV dominates HomePO (17%); (2) HomeIV is more costly and effective, and its ICER is less than or equal to the WTP, so it is cost effective (19%); (3) HomePO is more costly and effective, but its ICER is greater than the WTP, so HomeIV is optimal (4%); (4) HomeIV is more costly and effective, but its ICER is greater than the WTP, so HomePO is optimal (26%); (5) HomePO is more costly and effective, and its ICER is less than or equal to the WTP, so its optimal (6%); and (6) HomePO dominates HomeIV (28%).

Table 2 lists costs per episode and effectiveness of the four strategies under study. The model predicted that the average costs for oral outpatient management for adult cancer patients with low-risk FN would be lower than the costs for IV outpatient management, for the early discharge strategy and for in-hospital management. Considering quality of life, oral outpatient management yielded an average QAFNE of 0.65. This was inferior to outpatient IV and the early discharge option, but superior to the in-hospital strategy. Thus, our cost–utility analysis revealed that the oral outpatient strategy was cost saving, but less effective than its outpatient IV counterpart. The corresponding ICER was $10 186 per QAFNE. Both the early discharge strategy and in-hospital therapy were less cost effective than either outpatient strategies. Incremental cost-effectiveness ratios could not be calculated for the latter two treatment approaches as they were dominated strategies (Table 2). An ICE scatter plot comparing the two most cost-effective strategies (baseline=outpatient oral *vs* comparator=outpatient IV) is shown in Figure 2.

### Acceptability curve

**Figure 3:**
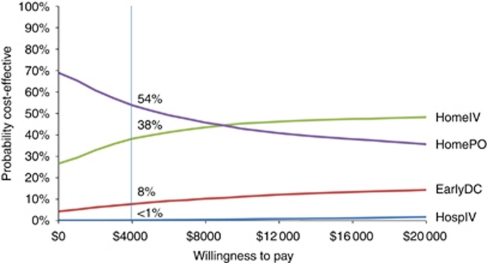
This chart presents the cost-effectiveness acceptability curve for the base–case analysis. The curves represent the proportion of simulations in which oral outpatient therapy and IV outpatient therapy, respectively, were the cost-effective option at various WTP thresholds. For example, at a WTP threshold of $4000 per quality-adjusted febrile neutropaenia episode (vertical axis), oral therapy was cost effective in 54% of the simulations.

At a WTP threshold of $4000 per QAFNE, oral outpatient treatment was cost effective in 54% of simulations, whereas outpatient IV was cost effective in 38% of the simulations. The early discharge strategy was cost effective in 8% and the traditional in-patient management was cost effective in less than 1% of the simulations. The results of the cost-effectiveness acceptability curve are shown in Figure 3.

### One-way deterministic sensitivity analysis

When tested for costs within the plausible range in a one-way sensitivity analysis, the model was sensitive to costs for in-patient stay per day (threshold >$2455), outpatient visit (threshold <$191) and home care nurse per visit (threshold <$49). The model was also sensitive within the plausible range to the following variables: duration of outpatient treatment (threshold <4.9 days); prolongation of therapy owing to complications (threshold >7.6 days); utility for outpatient IV (threshold >0.851); and utility for outpatient oral therapy (threshold <0.611). Beyond certain thresholds, dominance (less expensive and more effective) changed from the outpatient oral to the outpatient IV strategy. Importantly, there was not a single constellation when EarlyDC or HospIV became superior to the other two strategies.

## Discussion

This study suggests that outpatient strategies are the preferred approach to manage adult cancer patients with low-risk FN. Both outpatient oral and outpatient IV therapy are more cost effective than EarlyDC or entire in-patient management. This is, to our knowledge, the first model that addresses the question as to whether FN within the low-risk patient population is best managed in hospital or in an outpatient setting.

The probabilistic analysis in this study showed that in-patient care for FN was the least cost-effective strategy even when tested over a wide range of inputs/plausible values (in-patient care was cost effective in less than 1% of all Monte Carlo simulations). A likely explanation for the inferiority of the in-patient strategy in our model is its associated high costs. We found that the mean direct health costs were four-fold greater for in-patients as compared with oral outpatient management (Bennett and Calhoun, 2007). The economic burden for in-patient care in our study was higher than that in another recently published Canadian study (Lathia *et al*, 2010). On the contrary, one in-patient episode of FN in our model was, on average, less expensive as compared with recent cost estimates from the United States (Elting *et al*, 2008). We suggest that our single institution's estimates – given the plausible range/distribution used for the probabilistic analyses – permit broad applicability to other settings (Liou *et al*, 2007). In addition to higher costs, in-hospital care also seems to be associated with decreased health-related quality of life, at least at the aggregate level.

In contrast, our model was unable to consistently show the superiority of one of the two ambulatory strategies, namely outpatient oral and outpatient IV treatment. However, it appears reasonable that these two outpatient approaches present similar cost-effectiveness ratios. Moreover, having two comparable approaches in terms of cost effectiveness might allow patients to play a more active role in decision making in future, particularly if probabilities of treatment failure are similar between strategies, although other concerns such as feasibility may drive this decision.

Our cost–utility analysis has a number of important limitations. First, only limited data were available to estimate the base values in the model, leading to uncertainty with regard to the precision of the included event probabilities. To minimise this limitation, data obtained from a formal systematic review were applied across a wide range of values in our analyses. Second, we used VAS scores to derive health-related quality of life estimates for adult cancer patients with FN. To adjust for this limitation, we converted the VAS scores into standard gamble values by applying an equation developed by Torrance in the mid-1970s (Torrance, 1976; Torrance *et al*, 2001). Although not all studies have shown this technique to be valid, the credibility of our calculated standard gamble values has been supported by a recent study using the EQ-5D tool to elicit patients' preferences for in-patient management of FN (Torrance *et al*, 2001; Lathia, 2008). In the cited study, a mean health utility of 0.64 was calculated, which is almost identical to our converted utility estimate for patients treated entirely in hospital (mean standard gamble value 0.65) (Lathia, 2008). Third, the base–case analysis was performed using a hypothetical cohort. This might be advantageous in terms of external validity; however, the data are not easily applicable at the individual level owing to both covert and overt (e.g. preference) variability within this cohort. Fourth, the ICER of $10186 reported in our study together with the WTP threshold set at $4000 are difficult to interpret as FN episodes do not happen 12 times per year (to remember, $4000 per QAFNE was used as baseline value as this approximates a WTP threshold of $50 000 per QALY). Determining a reasonable ICER threshold for relatively short-term events remains contentious (Birch and Gafni, 2006). However, we suggest that an ICER of approximately $10 000 related to a transient episode that is unlikely to occur more than 2–3 times per year might be acceptable, at least for some countries. Nevertheless, it is difficult to define an acceptable ICER threshold for this decision-analytic model owing to the economic complexity associated with cancer treatment, including both the antineoplastic treatment itself and side effects other than FN.

Our study is important as it is the first quantitative attempt to compare different treatment strategies for low-risk FN in adult cancer patients. However, there are several important questions related to feasibility and individual preferences that need to be addressed in future research. First, are outpatient strategies feasible in settings such as lower-income countries and rural areas of high-income countries? The external validity of safe outpatient management has had limited evaluation outside the carefully controlled setting of RCTs. Second, further research is necessary to address the issue of preferences and medical decision-making. If efficacy and safety do not substantially differ between strategies, patients' preferences should impact on choosing the appropriate treatment setting and the way of drug administration (‘individualised patient care’). However, obtaining and interpreting patients' preferences for acute or non-fatal conditions can be problematic (Bala and Zarkin, 2000). ‘Bed-side’ tools that can be used to support the decision-making process would be highly desirable.

To conclude, our model suggests in adult cancer patients with an episode of low-risk FN, outpatient strategies are preferable to in-hospital treatment with IV antibiotics. Future research should focus on the feasibility of outpatient approaches in different settings, mechanisms to facilitate such an approach and the determination of patients' preferences.

## Figures and Tables

**Table 1:** Model parameter and distributions

**Parameter**	**Mean**	**Low**	**High**	**s.d.**	**Distribution**	**Alpha**	**Beta**	**Lambda**	**Source**
*Event probabilities*									
Probability of failure for hospital IV	0.082	0.048	0.109	0.01525	Beta	26.46	296.22		Hidalgo *et al*, 1999; Innes *et al*, 2003; Rapoport *et al*, 1999
Probability of failure for early discharge	0.175	0.152	0.208	0.014	Beta	128.73	606.88		Innes *et al*, 2003; Sebban *et al*, 2008
Probability of failure for outpatient IV	0.111	0.047	0.25	0.05075	Beta	4.14	33.17		Minotti *et al*, 1999; Rubenstein *et al*, 1993
Probability of failure for outpatient oral	0.189	0.095	0.208	0.02825	Beta	36.11	154.95		Hidalgo *et al*, 1999; Malik *et al*, 1995; Minotti *et al*, 1999; Rubenstein *et al*, 1993
Probability of readmission for early discharge^a^	0.5	0.25	0.75	0.125	Beta	7.50	7.50		Innes *et al*, 2003; Sebban *et al*, 2008
Probability of readmission for outpatient IV^a^	‘0.01	0	0.5		Triangular				Rapoport *et al*, 1999; Rubenstein *et al*, 1993
Probability of readmission for outpatient PO^a^	0.853	0.75	0.938	0.047	Beta	47.57	8.20		Hidalgo *et al*, 1999; Malik *et al*, 1995; Rubenstein *et al*, 1993
Rate of HCRI	0.006	0.0045	0.0075	0.00075	Normal				Kamboj and Sepkowitz, 2009
Relative risk of HCRI for outpatient IV	0.2	0.15	0.25	0.025	Normal				Simon *et al*, 2000
Relative risk of HCRI for outpatient oral	0.1	0.075	0.125	0.0125	Normal				Sewonou *et al*, 2002; Simon *et al*, 2000
									
*Utilities*									
Utility for inpatient IV	0.65	0.08	1	0.23	Beta	2.15	1.16		U
Utility for early discharge	0.72	0.16	1	0.21	Beta	2.57	1.00		U
Utility for outpatient IV	0.75	0.05	1	0.2375	Beta	1.74	0.58		U
Utility for outpatient oral	0.72	0	1	0.25	Beta	1.60	0.62		U
Relative reduction (factor) for utility if failure	0.8	0.6	1	0.1	Normal				Assumed
Relative reduction (factor) for utility if HCRI^b^	0.5	0.375	0.625	0.0625	Normal				Assumed
Relative reduction (factor) for utility if readmission	0.5	0.375	0.625	0.0625	Normal				Brown *et al*, 2001
									
*Costs*									
Costs per inpatient stay per day	2000	1000	4000	750	Gamma	7.11		0.0036	C
Costs of initial consultation	460	230	920	172.5	Gamma	7.11		0.0155	C
Costs for outpatient visit	320	160	640	120	Gamma	7.11		0.0222	C
Costs of home care nurse per visit	90	45	180	33.75	Gamma	7.11		0.0790	C
Costs of first-line IV antibiotics per day	100	50	200	37.5	Gamma	7.11		0.0711	C
Costs of second-line IV antibiotics per day	260	130	520	97.5	Gamma	7.11		0.0274	C
Costs of oral antibiotics per day	5	2.50	10	1.875	Gamma	7.11		1.4222	C
Relative increase in costs of antibiotics for HCRI^b^	1.5	1.125	1.875	0.1875	Normal				Assumed
									
*Time parameter*									
Duration of inpatient stay for hospital IV	6	3	12	2.25	Gamma	7.11		1.1852	Rapoport *et al*, 1999
Duration of inpatient stay for early discharge	2	1	4	0.75	Gamma	7.11		3.5556	Innes *et al*, 2003; Rapoport *et al*, 1999; Sebban *et al*, 2008
Duration of outpatient treatment for early discharge	4	2	8	1.5	Gamma	7.11		1.7778	Rapoport *et al*, 1999; Sebban *et al*, 2008
Duration of outpatient treatment	6	3	12	2.25	Gamma	7.11		1.1852	Rapoport *et al*, 1999; Rubenstein *et al*, 1993; Sebban *et al*, 2008
Prolongation of therapy related to complication^c^	6	3	12	2.25	Gamma	7.11		1.1852	Assumed
Time to complication^c^	3	1.5	6	1.125	Gamma	7.11		2.3704	Hidalgo *et al*, 1999; Innes *et al*, 2003; Malik *et al*, 1995; Minotti *et al*, 1999; Rapoport *et al*, 1999; Rubenstein *et al*, 1993; Sebban *et al*, 2008
Time to complication for early discharge at home^c^	1	0.5	2	0.375	Gamma	7.11		7.1111	Assumed

C: costs were obtained from local finance offices and the Department of Pharmacy at Princess Margaret Hospital in Toronto, Ontario/Canada.

U: utilities (visual analogue scale scores, converted into standard gamble utilities) were derived from 77 adult cancer patients at Princess Margaret Hospital in Toronto, Ontario/Canada.

a Conditional on failure of therapy.

b Healthcare-related infection.

c Complication=failure, readmission, healthcare-related infection.

*Note:* the probability of readmission for outpatient IV was 0% based on two published RCTs; however, to apply a reasonable distribution to this variable ( → triangular), a peak estimate of 1% (0.01) was chosen.

**Table 2:** Base–case analysis

	**HomePO**	**HomeIV**	**EarlyDC**	**HospIV**
Cost (mean)	$3470	$4183	$6115	$13 557
Cost (95% CI)	$1669–6564	$2001–7616	$2471–12 394	$4592–30 000
IncrC (mean)	—	$713	$1932	$9374
Eff (mean)	0.65	0.72	0.66	0.62
Eff (95% CI)	0.13–0.91	0.18–0.98	0.22–0.92	0.15–0.94
IncrEff (mean)	—	0.07	−0.06	−0.10
C/E (mean)	$5338	$5810	$9265	$21 866
ICER	—	$10 186	Dominated^a^	Dominated^a^

Abbreviations: CI=confidence interval; C/E=cost-effectiveness ratio; EarlyDC= treatment at home after an initial observation in hospital; Eff, effectiveness; Effectiveness=quality-adjusted febrile neutropaenia episode (rounded to 2 decimals); HomeIV=outpatient management with intravenous antibiotics; HomePO=outpatient management with oral antibiotics; HospIV=entire in-patient management; IncrC, incremental cost; IncrEff, incremental effectiveness; ICER, incremental cost-effectiveness ratio.

a Dominated refers to the finding that this strategy is dominated (e.g. both less effective and more costly than other strategies).

*Note*: All costs are given in Canadian dollars.

Options are ordered by increasing costs. Option 1 (HomePO) is the baseline reference to calculate incremental costs and effectiveness for option 2 (HomeIV). As option 2 is more effective than option 1, the former one is used as new baseline reference to calculate incremental costs (and effectiveness) for options 3 (EarlyDC) and 4 (HospIV). As options 3 and 4 are less effective and more expensive than option 2, they are both dominated by option 2.
