# Supplementary material for: Cost effectiveness of outpatient treatment for febrile neutropaenia in adult cancer patients
Source: Br J Cancer. 2011 Apr 5;104(9):1377–83. doi: 10.1038/bjc.2011.101 (PMC3101923; doi:10.1038/bjc.2011.101)
Supplement: Supplementary Data [file bjc2011101x1.doc]

**Supplemental Data A.** Details related to the construction of the decision tree

Building the decision tree required explicit variable information and assumptions related to the distinct pathways in the tree. The authors do not claim that the proposed model is the only reasonable approach; further, due to limited data available from the literature not all assumptions can be traced to published evidence but were rather felt to be clinically reasonable assumptions based on clinical experience and discussions with adult and paediatric oncologists and internists. However, probabilistic sensitivity analysis (PSA) was performed to account for uncertainty related to these limitations. For example, choosing different antibiotic drugs (or administration intervals) would only impact on cost factors (which are considered within the PSA) but not on event probabilities or utilities because these estimates were obtained independently.

- Outpatient intravenous (IV) antibiotics are given one to three times daily (administration through the use of pre-programmed infusion pumps is possible)
- On days of a clinic visit, outpatient IV antibiotics (AB) will be administered only once by the home care nurse
- The first outpatient visit after early discharge is 2 days later
- Patients treated in an ambulatory setting are seen every other day in the outpatient clinic
- Failure happens an average of 3 days after treatment initiation (and 24 hours after early discharge)
- Treatment failure in the early discharge strategy can only happen after discharge
- Treatment failure adds another 6 days treatment (on top of the initial 3 before failure) with associated increased costs
- Inpatient treatment failure activates second-line antibiotics (Meropenem-Vancomycin-Tobramycin = cost ABIV2)
- Outpatient IV treatment failure without readmission increases costs (cost ABIV2)
- Outpatient oral treatment failure without readmission leads to Meropenem IV monotherapy (cost = ABIV1)
- Anti-fungal escalation was not considered for low-risk febrile neutropaenia
- Patients in ambulatory care are also susceptible to healthcare-related infection (HCRI)
- The relative risk for a HCRI in the outpatient setting is 0.1 (oral) and 0.2 (IV), as compared to inpatient management
- The occurrence of a HCRI is not directly related to treatment success but to length of treatment duration
- HCRI happen at 72 hours after treatment initiation (and 24 hours after early discharge)
- HCRI are treated in the setting in which they occurred
- HCRI add another 6 days treatment (on top of the initial 3 before HCRI)
- HCRI increases treatment costs (baseline AB times 1.5) for specific treatment modification
- HCRI and treatment failure can be managed at the same time, thus, having both, adds a total of 6 days
- We assume independent utility reduction for treatment failure, readmission, and HCRI with factors of 0.8, 0.5, and 0.5

**Supplemental Data B.** Hypothetical scenarios

| **#1 INPATIENT MANAGEMENT**   - Admission in hospital with intravenous antibiotics - Blood testing 3x per week - If initial blood cultures are negative, patient is discharged after fever resolves and blood counts recover - Probability of complications leading to intensive care unit (ICU) admission is 2 in 100 patients - Probability of mortality is 1 in 100 patients |
| --- |

| **#2 INPATIENT MANAGEMENT + EARLY DISCHARGE**   - Admission in hospital with intravenous antibiotics - Early discharge within 24-48 hours with oral antibiotics (2 antibiotics given 2-3x per day) if blood culture is negative and patient is feeling well - Clinic visits for follow up and blood testing 3x per week - Once discharged, if patient is feeling unwell, they must return to the emergency department - Probability of re-admission is 5 in 100 patients - Probability of complications leading to ICU admission is 2 in 100 patients - Probability of mortality is 1 in 100 patients |
| --- |

| **#3 OUTPATIENT WITH INTRAVENOUS ANTIBIOTICS**   - No initial hospitalization, but blood culture and blood tests will be drawn - Discharge home with intravenous antibiotics - Home care nurse will come to patient’s home and administer 2 antibiotics once daily - Clinic follow up and blood testing 3x per week - If the blood culture comes back positive OR if fever persists OR if the patient becomes unwell OR the patient cannot tolerate oral antibiotics, the patient must return and be admitted to hospital - Probability of admission is 5 in 100 patients - Probability of complications leading to ICU admission is 2 in 100 patients - Probability of mortality is 1 in 100 patients |
| --- |

| **#4 OUTPATIENT WITH ORAL ANTIBIOTICS**   - No initial hospitalization, but blood culture and blood tests will be drawn - Discharge home with 2 oral antibiotics to be taken 2-3x per day - Clinic follow up and blood testing 3x per week - If the blood culture comes back positive OR if the patient becomes unwell OR the patient cannot tolerate oral antibiotics, the patient must return and be admitted to hospital - Probability of admission is 10 in 100 patients - Probability of complications leading to ICU admission is 2 in 100 patients - Probability of mortality is 1 in 100 patients |
| --- |

Legend Supplemental Data B: Outcome estimates (rates) for these hypothetical scenarios were derived from both randomized controlled trial and observational studies while the systematic review (to derive event probabilities for the decision-analytic model; see Table 1) was based only on randomized controlled trials. Thus, there are minor discrepancies of estimates between Table 1 and Supplemental Data B.

**Supplemental Data C.** Direct medical costs

| **Cost Factor** | **CAD** | **Source / Comment** |
| --- | --- | --- |
| **Hospital Stay per Day** | **2,000** |  |
| hospital stay per day | 2,000 | hospital fees, ultrasound, x-rays, diagnostic procedures; excluding physicians' fees |
| **Consultation** | **463.50** | **rounded to 460** |
| initial consultation (w/0 physician fee) | 238.00 | excluding diagnostic procedures/doctors' fees |
| initial consultation (physician fee) | 130.00 | PMH billing office |
| complete blood count | 8.00 | OHIP lab benefits |
| sodium | 2.50 | OHIP lab benefits |
| potassium | 2.50 | OHIP lab benefits |
| chloride | 2.50 | OHIP lab benefits |
| glucose | 2.50 | OHIP lab benefits |
| creatinine | 2.50 | OHIP lab benefits |
| urea | 2.50 | OHIP lab benefits |
| bilirubin | 2.50 | OHIP lab benefits |
| blood culture (aerob+anaerob) | 30.00 | OHIP lab benefits |
| urine culture | 10.00 | OHIP lab benefits |
| **Re-Assessment** | **323.50** | **rounded to 320** |
| re-assessment (clinic; w/o physician fee) | 238.00 | excluding diagnostic procedures/doctors' fees |
| re-assessment (physician fee) | 60.00 | PMH billing office |
| complete blood count | 8.00 | OHIP lab benefits |
| sodium | 2.50 | OHIP lab benefits |
| potassium | 2.50 | OHIP lab benefits |
| chloride | 2.50 | OHIP lab benefits |
| glucose | 2.50 | OHIP lab benefits |
| creatinine | 2.50 | OHIP lab benefits |
| urea | 2.50 | OHIP lab benefits |
| bilirubin | 2.50 | OHIP lab benefits |
| **Home Care Nurse Visit** | **90** | Community Care Access Center, Toronto |
| **Antibiotics (costs per day)** |  |  |
| Piperacillin/Tazobactam+Tobramycin | **100** | Department of Pharmacy, PMH |
| Meropenem+Tobramycin+Vancomycin | **260** | Department of Pharmacy, PMH |
| Ciprofloxacin+Co-Amoxiclav | **5** | Department of Pharmacy, PMH |

Legend Supplemental Data C:

OHIP, Ontario Health Insurance Plan; PMH, Princess Margaret Hospital, Toronto/Canada;
